# Maestro: An Orchestration Framework for Large-Scale WSN Simulations

**DOI:** 10.3390/s140305392

**Published:** 2014-03-18

**Authors:** Laurynas Riliskis, Evgeny Osipov

**Affiliations:** Department of Computer Science, Electrical and Space Engineering, Luleå University of Technology, Luleå 971-87, Sweden; E-Mail: evgeny.osipov@ltu.se

**Keywords:** wireless sensor networks, simulations, cloud computing, Amazon AWS

## Abstract

Contemporary wireless sensor networks (WSNs) have evolved into large and complex systems and are one of the main technologies used in cyber-physical systems and the Internet of Things. Extensive research on WSNs has led to the development of diverse solutions at all levels of software architecture, including protocol stacks for communications. This multitude of solutions is due to the limited computational power and restrictions on energy consumption that must be accounted for when designing typical WSN systems. It is therefore challenging to develop, test and validate even small WSN applications, and this process can easily consume significant resources. Simulations are inexpensive tools for testing, verifying and generally experimenting with new technologies in a repeatable fashion. Consequently, as the size of the systems to be tested increases, so does the need for large-scale simulations. This article describes a tool called Maestro for the automation of large-scale simulation and investigates the feasibility of using cloud computing facilities for such task. Using tools that are built into Maestro, we demonstrate a feasible approach for benchmarking cloud infrastructure in order to identify cloud Virtual Machine (VM)instances that provide an optimal balance of performance and cost for a given simulation.

## 1. Introduction

The emerging technologies and devices that will form the Internet of Things (IoT) will connect to the Internet via diverse network technologies, including Long Term Evolution (LTE), WiFi and Ethernet. Large enterprises, such as Cisco, Ericsson and General Electric have predicted that there will eventually be tens of billions of devices that are connected to the Internet and, thus, to one-another. This massive connectivity will impose new and previously unforeseen challenges in the design and maintenance of large-scale systems that incorporate loosely connected wireless sensor networks. These systems will, in turn, present unprecedented challenges in the design of backbone infrastructure and the demands placed upon it and will also place rigorous demands on future Internet infrastructure.

While these multi-billion device networks will be managed by many bodies, there is a need for understanding at the corporate level the effects that large-scale wireless sensor networks will have on infrastructure. In traditional network research, the impact of new protocols on infrastructure is evaluated using simulations. However, most of today's wireless sensor network (WSN) simulators are not designed to perform large-scale simulations or cannot be used to perform experiments using or in parallel with existing real-world systems. At present, most large-scale simulations are performed on high performance, distributed computing facilities, which require distributed schedulers and central coordination of the simulation. For example, one recent study [1] simulated over 360 million nodes by running ns-3 [2] on a computer with 4,400 cores delivering 52.8 teraflops. While systems of this sort have impressive capabilities, relatively few researchers have access to such resources. A small or medium-sized company or research group may find it almost impossible to access such facilities and conduct large-scale experiments.

Advances and vast industrial investments in cloud computing made Infrastructure as a Service (IaaS) available at a low cost. IaaS is an extremely scalable computing facility and now is accessible to a large scope of less well-resourced groups for performing large-scale simulations. Cloud computing has been used for scientific simulation previously; however, this adoption targeted specific simulation tools [3–6], adopting their internal particularities for usage with cloud computing.

Normally, even small-scale simulations are complex to setup and generate a large amount of data, which needs to be analyzed. As a result, computer communications research (computer communications are no exception to research fields that have been criticized; we simply limit our discussion in the scope of this particular field) based on simulations have been strongly criticized [7–9], because articles often fail to provide adequate detail on the setup of the simulations or to analyze the results obtained in sufficient depth. This lack of detail is often not due to shortcomings in the studies, but to the limitations on page numbers in scientific publications. Unfortunately, the lack of information can make it very difficult or impossible for other researchers to repeat, verify, use and build upon reported results.

This article presents a system named Maestrothat allows users to automate the running of both large-scale simulations and multiple simulations in parallel using IaaS platforms. Maestro was originally developed for use with Symphony [10], a framework for the virtualization, emulation and simulation of wireless sensor networks. Symphony enables simulations to be performed using the same code base as would be deployed on a real wireless sensor node. Moreover, it preserves the execution timing of the real system by performing hardware modeling based on measurements of real hardware. In this article, the Maestro framework for the automation of large-scale simulations is presented in a generic manner and is decoupled from the specifics of the particular simulation environment that is showcased.

Additionally, a cloud-based workflow is proposed, and a method is presented that addresses the questions that have been raised about the credibility of simulations in computer communications research. The workflow of this method is general enough to be adopted in other fields that rely heavily on simulations.

Importantly, these systems could potentially support a more open research community, allowing both results and the details of a simulation's set-up to be shared with other researchers in a practical and straightforward way.

Finally, the Amazon Web Services (AWS) infrastructure is benchmarked using a tool provided by Maestro to illustrate the process used to identify the optimal type of instance in terms of balance between performance and cost for a specific simulation.

The article is organized as follows: Section 2 discusses related previous work in the context of this article. Section 3 provides an introduction to the Symphony framework to facilitate the presentation of the framework. Section 4 describes the architecture of Maestro and the workflow for its configuration and usage. Section 5 describes the benchmarking tools provided with Maestro and the results of a benchmarking study performed on Amazon's AWS cloud infrastructure. Section 6 showcases the setup of large-scale WSN simulation using Maestro and Symphony and discusses the consequences of the choices made during the setup process. Finally, concluding remarks are presented in Section 7.

## 2. Related Work

In this section, we review existing approaches for large-scale simulations for wireless sensor network simulations. Simulators in this area range from pure synthetic simulators, where only pseudo implementation can be tested, to instruction-level simulators for running emulated nodes in the simulator. While different levels of system abstraction are targeted by different approaches for the implementation of the simulator, we particularly focus on aspects of simulations in real time, at large scale and the interoperability with real systems and or other simulators.

To improve the scalability of network simulations, several parallel discrete simulators have been developed. The traditional approach for implementing parallel simulations is to create a custom simulation software with a scheduler supporting parallelism. Examples of such a simulator are Global Mobile Information System Simulator (GloMoSim) [11] and ns-3 [2,12], among others. The advantage of such an approach is the efficiency of execution of such an implementation; the parallel scheduler is tailored for a specific simulation environment. However, new models must be implemented specifically for such a system, thus requiring a significant amount of effort. The second approach includes interconnecting multiple copies of the simulator or different simulators. This approach allows the reuse of models and software from different simulators in one system [13]. This approach is, for example, utilized by Framework for Parallelization of Network Simulations (PDNS) (based on ns-2) [14], GTNetS [15] and SimGrid [16]. While the first approach requires super computers enabled with Message Passing Interface (MPI)programing interfaces, the second approach utilizes computer clusters.

The simulation approaches described above are utilized mostly for synthetic load in computer networks. The emerging Internet of Things and cyber-physical systems e.g., wireless sensor networks, most commonly consist of many components, such as sensory data, networking, backend system, data mining, etc. Therefore it is often impossible to obtain theoretical or analytical results to compare the performance of algorithms targeting such holistic systems. One possibility is to conduct large numbers of experiments on a real system. While this is possible on a tightly-coupled system, it is infeasible on loosely coupled WSN systems. Consequently, one must rely on simulations, which enable reproducible results and also make it possible to explore wide ranges of platform and application scenarios. Therefore, it is important that simulators are as close to the real code base and the real system as possible.

When it comes to simulating wireless sensor networks, the scalability is often limited by the simulator supplied with the operating system. For example, Contiki [17] is supplied with Cooja [18] and TinyO [19] with TOSSIM [20]. While, Cooja allows users to choose the desired level of abstraction in simulation ranking from pure “java node” to emulated instruction level simulations [21], TOSSIM only offers one level of abstraction, and the state of the node is represented as an entry in an event table. Both simulators scale to 20,000 nodes, but Cooja can handle only up to 100 emulated nodes (depending on the underlying hardware characteristics of the PC).

In the context of this article, none of the above approaches offers the same simplicity for large-scale WSN simulations with a real code base. The simulation scaling approach taken by Maestro is feasible for a set of WSN applications, where the node clusters have no interdependence and can be simulated separately. While the previously mentioned simulator has different performance characteristics (see [22] and the reference within), it is important to emphasize that Maestro is a tool enabling vertical scaling of most of the simulators described above by utilizing advances in cloud technology and is not a simulator itself.

## 3. Symphony

**Figure 1.**
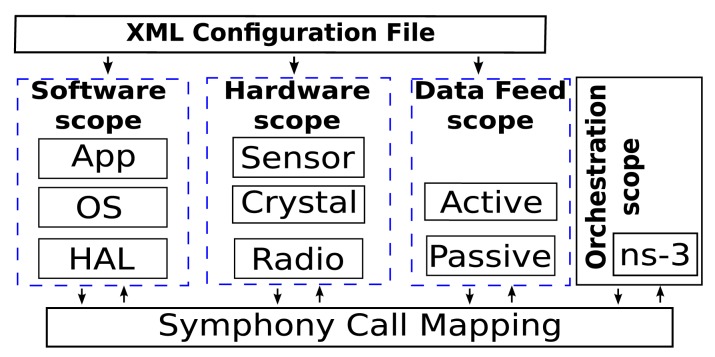
Architecture of the Symphony framework. Programming scopes are developed independently and later configured using XML file which describes nodes model. The scenario of simulation is created in ns-3 traditional way.

We present the approach to orchestrate large-scale simulations and the Maestro toolbox using an ns-3-based simulation framework as the platform to scale. Symphony [10] has several features that make it particularly useful for large-scale WSN simulations. First, it can run simulations using the actual code that would be deployed in the real network and provides tools for the precise emulation of real hardware. Second, its data feed model allows sensors to receive raw sensor data for processing and to then transmit the processed data to a real backend. Furthermore, Symphony incorporates ns-3 to facilitate scenario creation and the performance of network simulations that can model channels, such as LTE, WiFi and Ethernet and connect to real backends. Figure 1 illustrates the core architecture of Symphony and its four programming scopes. The software scope deals with the mapping of function calls to the underlying hardware scope. The level of abstraction is configurable, and the scheduler of the underlying WSN OS is preserved. In the Figure 1, three abstraction levels are depicted. App - being the highers abstraction level and simulating on the application level, OS - being middle tier and emulating on the operating system level, HAL—is the lowest level of abstraction emulating on the Hardware Abstraction Layer. The hardware scope consists of a clock and a series of models for hardware components, such as radio devices and sensors. These hardware models ensure that the application code is executed on a device-specific time scale. The data feed scope contains mechanisms for either actively or passively feeding data to the relevant software handlers of a specific sensor node. The orchestration scope is implemented on top of the general purpose network simulator, ns-3. It is responsible for integrating all of the other scopes with the sophisticated communication models and the ns-3 event scheduling engine to create a holistic simulation environment. All of Symphony's operational scopes are parameterized using a single XML configuration file.

### 3.1. Models of Operating Scopes and Profiling Principles

**Figure 2.**
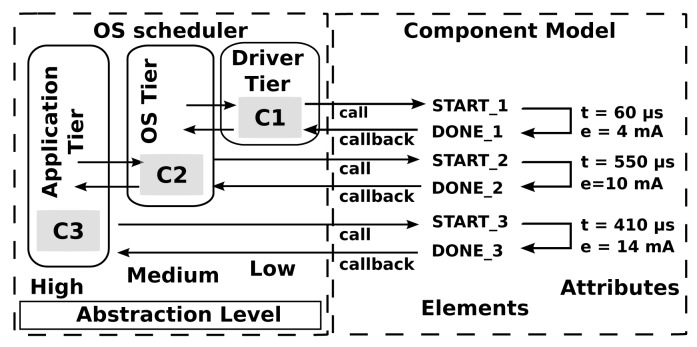
Model configuration using XML and hardware models.

In Symphony, nodes are simulated using a set of models that provide homogeneous descriptions of the behaviors of both hardware and software components in terms of execution times and energy consumption. Figure 2 outlines this approach to modeling. The figure shows three components, *C1, C2*, and *C3*, which describe the functionality at different levels of granularity. *C1* is the lowest level (*i.e.*, hardware) component and may represent something like a radio device and the associated driver. It performs the primitive operations of sending and receiving bytes. The *C2* component represents a higher level of functionality, such as a function that queues packets, inspects and modifies their headers and then transmits them onwards. Finally, *C3* represents the highest level software components, such as functions that accept packets, encrypt and decrypt them and perform application-specific operations before transmitting them onwards. The level of granularity in the simulation can be configured by the user. For example, it is possible to perform system-level experiments using only application-level components, or, at the other extreme, to focus on low-level operations using driver-level models. Simulations of the latter sort are particularly useful for very fine-grained debugging.

The component models describe the component's time and energy behavior when it is called via a call and when it returns control to its caller via a callback .

The component models also describe the properties of the callback. These include information on the return type and the input parameters of the function. The time and energy values are determined by measuring the time required for the device of interest to perform a specific operation and the energy consumed when doing so.

### 3.2. Software Scope

Symphony does not make any short cuts when simulating WSN functionality: a real operating system is virtualized, and its execution model is preserved. In essence, Symphony intercepts calls at the desired level of abstraction and delays their execution according to the profile of the corresponding component. Symphony can be used to perform simulations on three tiers: low, medium and high. Higher tiers correspond to increased granularity in the simulation and, therefore, more complexity.

The application tier is used to perform system-level simulations and represents the highest level of abstraction available in Symphony: only the highest level calls are passed through. Therefore, when the node is profiled, it is only the effect of these calls that is measured. This tier yields the fastest simulations.

**Figure 3.**
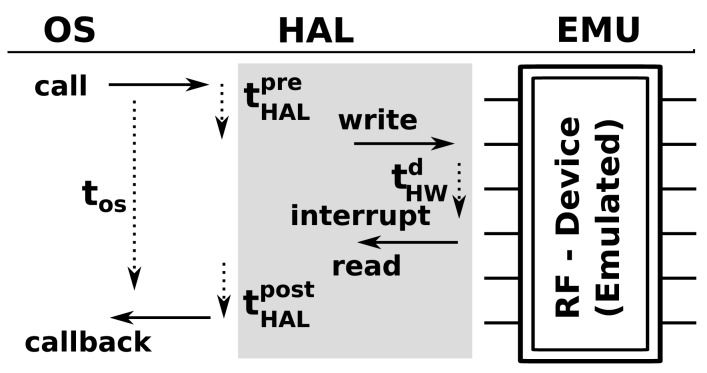
Call propagation between different abstraction levels and the emulated hardware. The OS represents operating system level, HAL shows Hardware Abstraction Level, and EMU depictures the emulated hardware device, in this particular case radio transceiver (RF).

When a function, such as AMSend, is called from the application tier, it will generate a fork with several calls that propagate from the OS tier down to the hardware level. These calls and callbacks will do things, such as initialize the relevant hardware components and handle retransmission or acknowledgment of the original call. From the perspective of the OS tier, these operations will consume a certain amount of time, *t_OS_*. However, in reality, there will be many pre- and post-call code executions associated with each call, as shown in Figure 3 (HAL). In cases where these hardware abstraction layer (HAL)-level details are important, profiling must be done at the HAL level. This will entail measuring the execution time and energy consumption for each operation of interest. It is the most accurate tier of simulation, but also the most computationally intensive and will significantly reduce the number of nodes that can be virtualized per core relative to the other tiers.

### 3.3. Hardware Scope

Hardware interrupts and calls are emulated by tapping into the hardware abstraction layer (HAL) of the WSN OS. As shown in Figure 3, when an operating system makes a call to a hardware element (in this case, a call to a radio transceiver to transmit a message) in Symphony, the call is dispatched to the appropriate hardware model. Essentially, the device model is a piece of software that mimics the behavior of the real hardware component. Technically, all of the modes used in Symphony are inherited from the *ns3::Object* class and parameterized according to the appropriate XML configuration file.

### 3.4. The Data Feed Scope

One of the common shortcuts taken by researchers when conducting simulation-based investigations into the performance of networking functionality in wireless sensor networks is to work at a level of abstraction that does not require the consideration of real sensory data. It is often assumed that the sensory data is instantly available for transmission and that its acquisition does not in any way interfere with the sending-receiving processes. In addition, protocols are often stress tested using synthetic cross traffic.

However, in reality, the flow of sensory data through wireless sensor nodes has significant effects on the performance of all of the network's software components. In brief, before it can transmit the data, the sensor must warm up, sample the environment, pre-process the data and packetize it. All of these operations take time. Moreover, if the data handling component is not implemented correctly, it may prevent the execution of the sending/receiving procedure and, thereby, violate the logic of the protocol being studied. Symphony has a native tool for addressing this issue in the form of its Data Feedscope, which makes it possible to work with either pre-recorded real data traces or data that are fed into the Symphony node in real time from real hardware.

**Figure 4.**
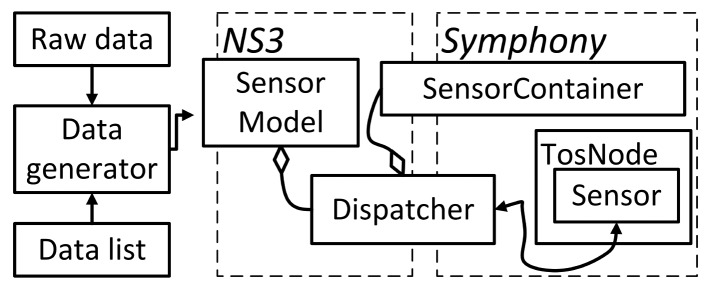
The architecture of the Data Feedscope that supports the sensor model.

The architecture of the Data Feedscope is shown in Figure 4. Symphony can handle both pre-recorded raw data and data supplied in real-time from an external generator or via a numerical data list. The data generatorinterprets and retrieves data from specified locations. Its main purpose is to hide the specific method used for data retrieval and make the sensory data available to the sensor modelin a generic format. Two sensor types are supported by the model: active sensors, which issue interrupts when data becomes available, and passive sensors that release data in response to periodic polling by the OS. The sensor modelmakes the data available to the operating system of the sensor node with delays that are specified in the appropriate configuration file. For active sensors, the model will issue an interrupt according to timestamps provided by the data generator. When the OS issues a call for a data reading to a passive sensor, the sensor model will look up the data in a list using the current time as a key.

## 4. Maestro: A Tool For Orchestrating Simulations in Clouds

Wireless sensor networks have applications in several areas, including intelligent homes, facility management, machine surveillance, preventive maintenance and industrial automation. One common denominator for most WSN use cases is the flow of data from the sensors to a backbone server. Wireless sensor networks usually communicate via gateways using various radio technologies, such as WiFi, LTE or Ethernet. This data can subsequently be accessed by end users via the web or some other interface. Certain WSN setups can be regarded as clusters of nodes, each of which forms an “island”. Such setups lend themselves particularly well to large-scale simulation in clouds, because individual islands from the overall network can be simulated on separate instances (Since, for the practical implementation of Maestro, we used the Amazon AWS cloud, throughout the article, we will use AWS-specific terminology and generic terminology interchangeably. Thus, “instance” refers to a running Linux virtual machine, Amazon Virtual Machine (AMI) , virtual machine image) and, just as in reality, connected to one-another via a backbone infrastructure. This approach makes it possible to simulate the entire application using a large number of sensors and actuator devices in a wireless sensor network at once and, thus to test and verify the functionality of the entire system. Maestro was designed with such scenarios in mind, but it can also be used to execute multiple serial simulations in parallel. This is especially useful for validating results obtained in previous simulations and can dramatically reduce the overall time required to perform and validate simulation-based studies.

**Figure 5.**
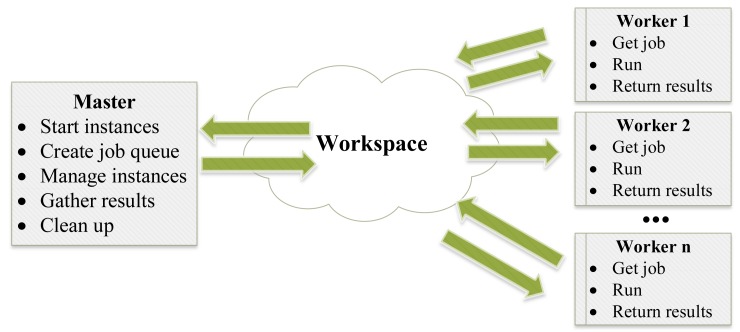
A schematic illustration of Maestro's general design.

Maestro is based on a master-worker system architecture, as shown in Figure 5. The master reads a configuration file and then starts workers and creates the workspace accordingly. The workspace consists of the tools and protocols that enable the automated control of workers, the execution of jobs and the gathering of simulation results. Maestro also provides tools for automated simulation performance benchmarking with respect to the usage of Central Processing Unit (CPU) and memory resources. These benchmarking tools are used to identify the most appropriate instance for specific simulations and to monitor the execution of the simulation. The remainder of this section discusses Maestro's architecture in more detail and the process of its initialization when setting up large-scale simulations.

### 4.1. The Architecture

**Figure 6.**
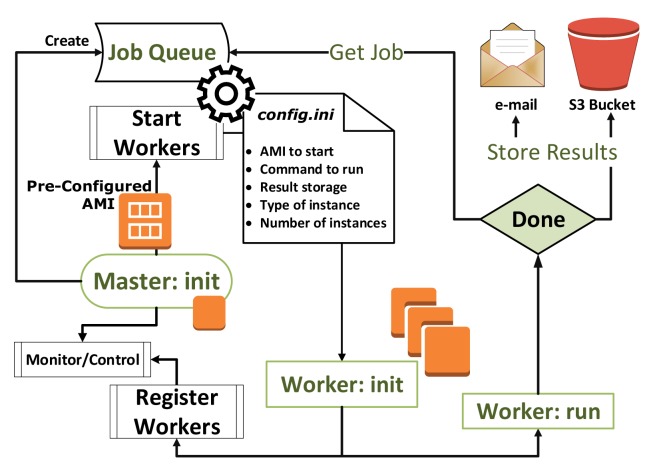
Maestro's system design and workflow.

The architecture of Maestro and its workflow are outlined in Figure 6. The core of Maestro consists of a Master instance that initializes simulation resources according to specifications provided in the configuration file. The Master node has three key components: the Client Server, the Cloud Commander and Result Bucket.

The Client Server is a service that allows the simulation to be accessed and started up remotely. The client (user) can submit a configuration file using a local command line application. When the configuration file is received by Maestro, it starts other services and keeps the client notified about the simulation's progress.

The Cloud Commander (CC) is the core service of Maestro. When activated, it reads the configuration file and allocates computing resources accordingly. During the initialization phase, CC will start the specified number of instances of a particular virtual image (AMI in the case when AWS is used).

It will then wait until these instance have booted and become reachable. Once this has happened, CC will create a pool of threads that will establish two communication channels for each instance. The purpose of the first channel is to command and control the Worker instance. This channel is primarily used during the startup phase and at the end of the simulation, when the results are collected from the Worker. During the startup phase, a simulation job from the Job Queue will be sent to the Worker instance via the allocated thread. Once the simulation has been completed, this thread will gather the results from the instance and store them locally. It will then wait for the Job Queue to become empty, at which point it will terminate the instance and shut itself down. The properties of the configuration file and the commands that can be issued to Worker instances are detailed below. The secondary communication channel is used to monitor the health and status of the instance. A simulation may have non-deterministic termination conditions; for example, it may be required to continue running until a some predefined statistical condition has been satisfied. In such cases, if a simulation continues to run for a long time, the lack of terminal access can make it hard to determine whether this is because the condition has not yet been satisfied or because the simulation software has crashed or become deadlocked, *etc*. Moreover, infrastructural failures can generate “zombie” instances, *i.e.*, partially break the simulation and, thus, may affect the intended scenario and expected outcome. One way of determining the status of an instance is to monitor its CPU usage, memory allocation and Input/Output (I/O) performance. However, a high CPU usage or memory allocation is not necessarily indicative of a healthy instance, as discussed above. Therefore, the secondary channel is used to perform additional Worker health checks. If a Worker instance does not respond to these checks appropriately, it will be marked as a zombie and terminated, after which the thread will exit.

Once the Job Queue has emptied and all of the threads it spawned have terminated, the thread pool will exit and CC will activate Result Bucket. The purpose of Result Bucket is to process and store raw data generated during the simulation. To this end, it can apply user-provided scripts to the simulated data. The results are stored in a user-specified location and can also be emailed to specific destinations, made available via FTP server, or a web server or uploaded to a cloud storage facility, such as Amazon S3.

### 4.2. Worker Configuration Workflow

Before the simulation can be run, the Worker instances must also be configured. This section describes the steps of the Worker instance configuration process that are independent of the simulator to be used. The Worker instance is started by the CC service, which opens two Transmission Control Protocol (TCP)service sockets for incoming connections when booted up. The first of these is used to receive the simulation job and the second to monitor the health of the simulation. After the simulation job has been received, the worker service will start the specified number of simulations on the selected instance and capture the output of the simulator. At the same time, the health monitoring service is started. This service continuously checks for zombie simulation processes and monitors the status of the simulation. Instance health data are transmitted to the Master node via the second channel. Once the simulation has finished, the results are gathered and transmitted to the Master Server, and the worker is placed into standby mode to await a new job.

**Figure 7.**
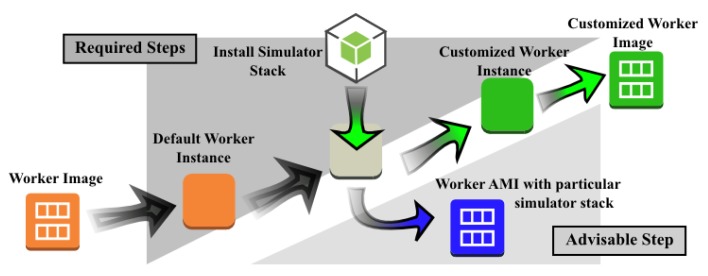
Workflow for setting up a custom Maestro Worker.

A virtual machine image that is to be used as a Maestro Worker must be prepared as shown in Figure 7.

The configuration of the Worker instance involves four steps. First, the user starts an instance using a default worker virtual machine image that provides a minimal set of tools and software packages that are required by the Worker services described in the preceding subsection. Second, the user logs in into the instance via Secure Shell (SSH)to access its terminal interface and install the simulation stack, tools and libraries that are needed to build and operate the simulator. At this point, it is advisable to create a new virtual machine image of the instance before configuring it for a specific scenario and simulation case. Third, the user configures the simulator and scenario. It is sensible to perform a test run at this point to ensure that the system is working as intended. After the simulator has been configured successfully, the user creates a new customized worker virtual machine image, thereby completing the worker configuration process.

After the customized worker instance has been created, the user must create a Maestro configuration file according to the specification provided in Listing 1.


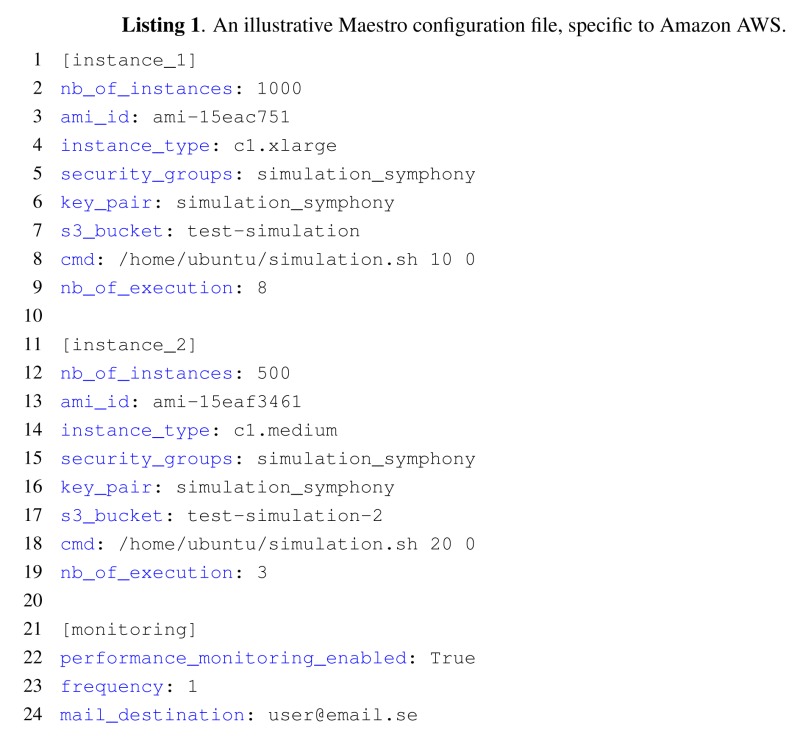


The Master accepts multiple instance configurations (limited only by the number of threads and socket ports that can be created in the operating system that is being used), so it is possible to run several distinct simulations at once or a single very large simulation or some combination of the two. The configuration file must specify the desired number of instances (nb_of_instances), the ID of the prepared virtual image (ami_id) and the type of the instance (instance_type). In addition, it should provide security credentials (security_groups,key_pair), state how the results should be stored (s3_bucket) and specify a simulation command to execute. The number of parallel executions per instance is controlled by the nb_of_execution variable in the example listing. This variable is useful when the simulation is to be run on instances with multiple physical cores, but the simulator uses only one core. The configuration file for *instance_1* would start 1,000 instances of the c1.xlarge type, with each instance launching eight parallel simulations, whose results would be stored in the *S*3 bucket.

### 4.3. Benchmarking Tools Provided by Maestro

Each simulation scenario and tool has distinct performance requirements and run-time behaviors. While some simulators run in a single thread, others will spawn multiple threads or allocate several cores. The number of simulations that can be run in parallel will depend on the simulator that is used. Consequently, before performing a large-scale run, the user should benchmark the available instance types and identify that which is best suited to the task at hand in terms of resource allocation and cost. This subsection describes the benchmarking tools that have been incorporated into Maestro; Section 5 provides more details on the benchmarking process and provides an illustrative example.

**Figure 8.**
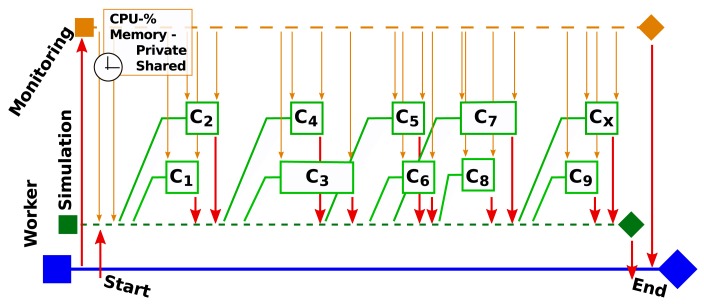
Scheme showing the sequence of events when a Maestro Worker (shown in blue) running a simulation thread that spawns multiple child threads is started. The simulation thread (in green) is initialized, after which the monitoring thread (in orange) is started. The simulation thread is then started and spawns child threads (C_1_-C*_x_*) as it proceeds, each of which is monitored individually by the monitoring thread (indicated by the orange arrows). The transfer of data between threads is indicated by the red arrows.

Figure 8 shows a representative simulation scenario (in the ns-3 simulator) in which the simulator spawns child threads to perform its tasks. When the worker is started, it initializes the simulation thread (*S_t_*) and then starts the monitoring thread (*M_t_*). Once this has been done, the simulation thread is started. *M_t_* then regularly reads information on *S_t_* provided by the platform utilities. This information includes the process IDs (PIDs) of the child threads, as well as performance metrics for *S_t_* and its children, such as the CPU usage and the amount of allocated private and shared memory for each thread. These data are returned to the owner thread (which would be the worker in a benchmarking scenario).

The benchmarking tools are also used to monitor the health of the worker instance. In this case, they are started by the health monitoring thread and configured in a different way in order to minimize their resource consumption.

### 4.4. A Note on the Use of Maestro with Large-Scale Simulators and for the Parallel Execution of Serial Simulations

It is important to note that Maestro is a non-intrusive tool that can be used both to automate the running of large-scale simulations and to execute multiple serial simulations in parallel. However, while it provides tools for experiment deployment, it does not synchronize the simulator's simulation scheduler, nor can it be designed to interfere with the set up of a specific run of the simulation. Therefore, when performing large network simulations using Maestro, the user must take care to set the simulation scenario up appropriately. The Master node can be used to distribute the network addresses of the started instances in cases where a dynamic scenario setup is required.

### 4.5. A Note on the Synchronization of Large-Scale Simulations

When large-scale simulation is performed, the number of instance can exceed current availability in a particular data center or introduce variation in the boot time of the instance. Thus, it is challenging to synchronize the large number of instances used in the simulation. To overcome this issue, Cloud Commander waits, prior to the simulation start, until all of the instances are booted and have registered. Amazon AWS provides the means for accessing state attributes of the instance, which makes the synchronization easier. When the instance is booted, the IP address is assigned to it and can be retrieved by queering Amazon Web Services (AWS) Application Programming Interface (API). Therefore, the Master node waits until all instances have booted and have been assigned an IP address. Preceding this discovery, a communication channel for a monitoring instance is established, and the distribution of Job Queue starts. Being non-intrusive and a generic tool, Maestro leaves it up to the user to synchronize the simulation if the simultaneous start-up is not sufficient.

### 4.6. A Note on Implementation

Maestro has been implemented in Python in combination with Boto, a Python package that provides interfaces for accessing AWS. Boto is also compatible with Eucalyptus, a free and open-source software package for building AWS-compatible cloud infrastructure. Our solution can thus be deployed either on AWS or on a private cloud infrastructure. Both the Master and Worker instances run on Ubuntu 13.04 images. The master images are fully pre-configured and can either be controlled using a local initialization file or via remote client extension. The only pre-configured tools on the worker instances are those required to join the workspace and listen for incoming connections from the Master node.

## 5. Benchmarking EC2's for Simulations

Maestro makes it easy to deploy either large-scale simulations or multiple replicate instances of a smaller simulation. Cloud computing provides computational facilities that are capable of vast horizontal scalability and also significant vertical scalability. However, while horizontal scaling is relatively straightforward to achieve (one need only decide on a number of instances to use), vertical scaling is more complex. In particular, vertical scaling requires the careful selection of an appropriate instance type to match the task at hand. This means that it is important to benchmark the performance of the available instance types using a workload similar to that which will be used in the simulations. This section describes a representative benchmarking study conducted on Amazon's EC2cloud computing platform, which is a component of the AWS cloud system. In addition, a general procedure for identifying and selecting optimal instance types for a given simulation is outlined. The AWS system provides seventeen types of Linux architectures. Table 1 outlines the properties of twelve of these architectures. Light gray rows indicate instance types that were benchmarked in this work. It should be noted that some of these instances enable the creation of an optimized Elastic Block Store, which increases their I/O throughput.

We performed benchmarking experiments with the Symphony framework, which uses ns-3 for orchestration and network simulation. All of the benchmarking results presented below were obtained by performing 100 separate simulation runs using each combination of instance type and framework settings and averaging the results of these hundred runs. When analyzing the results of the benchmarking study, it should be noted that the results obtained will depend on the precise details of the benchmarked setup (e.g., the numbers of nodes used and tests run). In addition, the results obtained may not be representative of those achieved using different simulators or even the same simulator with a different set of scenarios.

The Symphony framework was benchmarked using three different commands: *waf -j1 build, waf build* and *test.py*. The *waf -j1 build* command builds the simulation framework, but limits it to single-threaded operation. The second command, *waf build*, builds the simulation framework with a number of processes that is determined by the waf implementation. Finally, *test.py* is a simulation setup that performs 100 simulations in a row.

The computational power of the instances provided via Amazon's virtualization services is specified in ECUs. ECU stands for EC2compute unit and is a relative measure of integer processing power. This metric was introduced to provide a consistent measure of a system's computational performance as the underlying infrastructure evolves.

**Figure 9.**
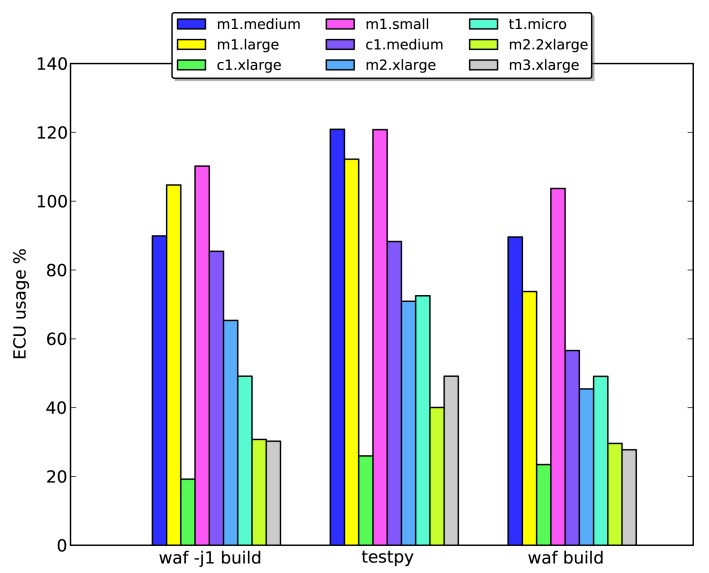
ECU usage for the different instance types running each of the three benchmarking tests.

Figure 9 shows the load on selected instance types as a percentage of one ECU when running the three benchmark tests. It is clear that the ECU performance for m* instances is somewhat inconsistent (recall that m instances have large amounts of memory), since in several cases, their load is in excess of 100%. However, over all three tests, they seem to be the most resource-efficient options. Resource efficiency is an important parameter, and one key goal of benchmarking is to identify and select instances that will be maximally utilized by the simulation.

### 5.1. A Note on Benchmarking

**Figure 10.**
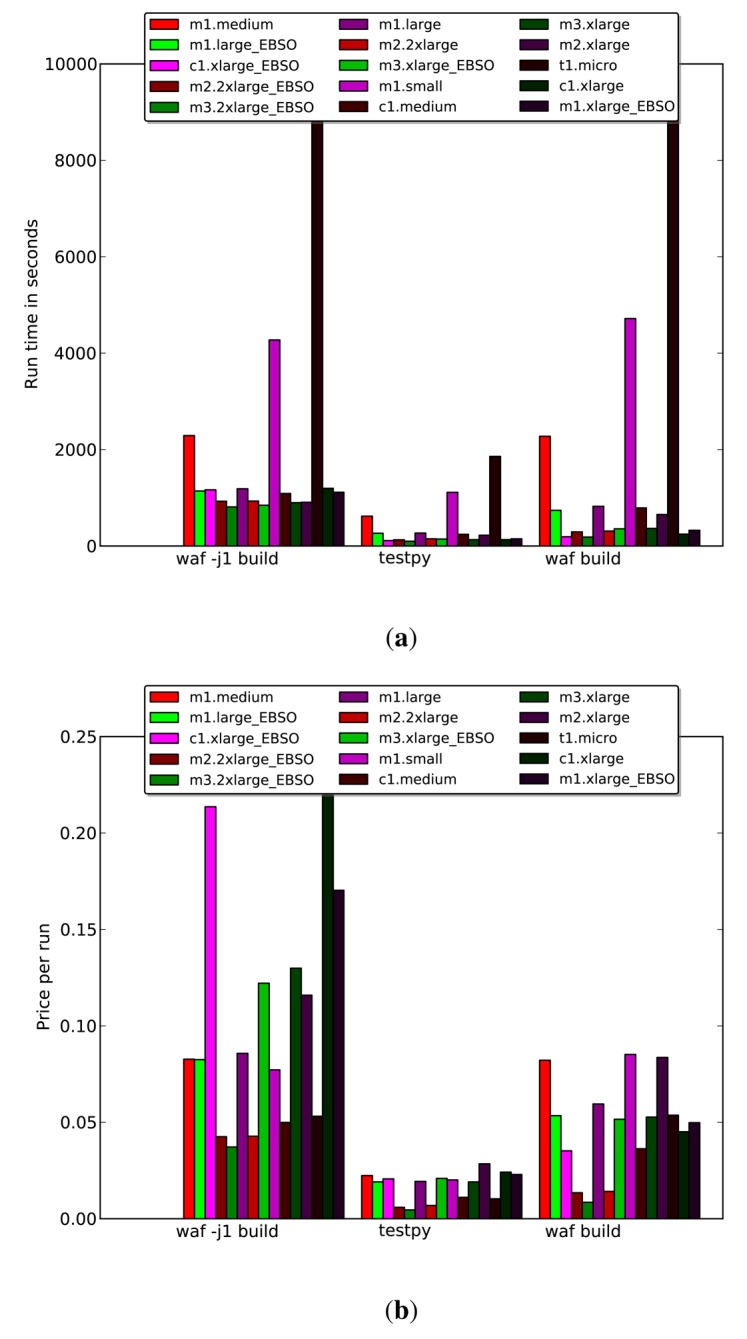
Simulation benchmarking results on Amazon Web Services (AWS). (**a**) Run times for the three benchmarking tests on different instances; (**b**) simulation times as a function of the number of nodes used when running the three benchmarking tests.

In subsequent benchmarks, we examined an additional set of instances with consistent disk read/write (I/O) throughput values. These instances are indicated by the suffix _EBSO. Figure 10a on each instance type. The imposition of a fixed I/O throughput did not have any appreciable effect in the benchmarking tests.

Figure 9 clearly shows that while the m1.-small, -medium and -large instances had the best levels of ECU utilization, they were outperformed by even more memory heavy instances, such as m3.2x large, with the _EBSO variants being slightly faster than the instances without consistent I/O throughput. Somewhat surprisingly, these instances outperformed the more computationally powerful units from the *c* series. This may have been because they have more and faster memory, which would have reduced the degree of content switching during the simulations. An important metric to consider when selecting instances for large-scale simulations is the cost per run, which is shown for the tested instance types in Figure 10b. In this case, the test runs were short. However, we assume that under normal conditions, the runs would proceed for more than one hour (which is the minimum billing period). We therefore calculated the cost per unit time for each instance type rather than comparing billed times. Surprisingly, the most expensive high memory instance (m2.2x large) offered the lowest running costs of those tested.

We conclude that there are three important metrics to consider when benchmarking instances to determine their efficiency at running a given simulation. While ECU utilization is an important factor, the price per run and runtime are more useful when seeking to identify the most appropriate instance for a given simulation scenario.

## 6. A Million Node Simulation of a Holistic WSN-Based System

**Figure 11.**
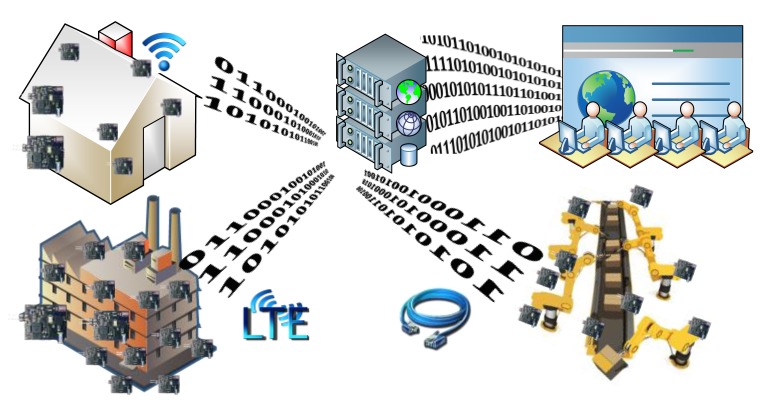
Wireless sensor network (WSN) usage in different scenarios.

As discussed in Section 3, wireless sensor networks have diverse applications, but generally require a flow of data from the sensors to a backbone server. As shown in Figure 11, wireless sensor networks usually transmit data to a gateway using various radio technologies, such as WiFi, LTE, Ethernet, and so on. End users can then access these data from the gateway via web-based interfaces or other means. The WSN is, thus, clustered into smaller ‘island’ networks. Because of this clustering, WSNs lend themselves particularly well to large-scale simulations in clouds, because individual islands can be simulated on separate instances that are connected to some kind of backbone infrastructure, exactly as occurs in a real WSN. By taking this approach, it becomes possible to both simulate the wireless sensor network and simultaneously test and verify the system as a whole.

### 6.1. From Deployment to Simulation: A Showcase Scenario

**Figure 12.**
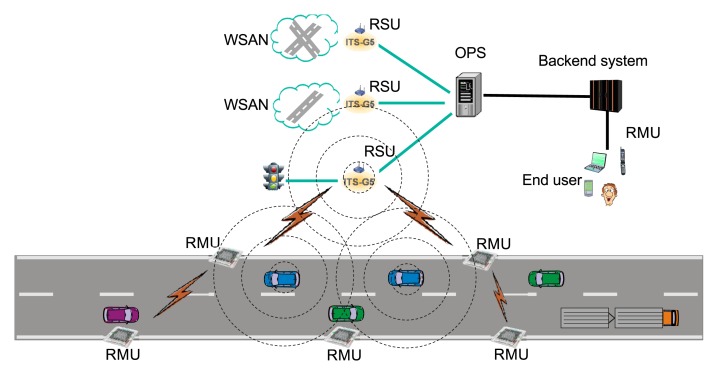
Simulation scenario representing an intelligent transportation system with road side marking units (RMUs) placed along the route. RSU, road side unit; OPS, open platform server.

We have previously introduced the road surface network (RSN) architecture [23] as a building block for intelligent transport systems. The proposed RSN architecture is illustrated in Figure 12. It consists of three principle entities: road marking units (RMU), road side units (RSU) and an open platform server (OPS) that will enable the incorporation of new RSN services in larger Intelligent Transportation System (ITS)systems. RMUs are autonomous on-road devices that may work independently or cooperatively to perform sensing and actuating tasks. RMUs are capable of wireless communication with RSUs and can also communicate with one-another, thereby forming a wireless sensor network. RSUs are the gateway nodes that convey data between RMUs and the ITS backend system. The open platform provides a set of open interfaces that connect RMUs to a backend ITS and front ends.

A small test site was constructed by researchers at Luleå University of Technology [24] to evaluate the performance and functionality of the RSN technology. The test site contained 14 active communication and sensing nodes, as well as actuator nodes. While 14 nodes is a relatively small number in the case of major ITS application, it is a practically useful number of sensors connected to a gateway given the range of the underlying radio technology. All of the nodes were deployed on roads leading towards a roundabout. The sensing nodes were equipped with accelerometers and magnetometers, whose readings were used to determine the type, speed and position [25–27] of cars traveling along the road. The actuation nodes were equipped with LEDs and had radios for communication. The purpose of the test system was to detect approaching cars and switch on running lights around the roundabout in response to their presence. During the system's deployment and testing, a number of issues and questions arose that prompted the development of a framework for its large-scale simulation. Each sensor generates 1, 200 bytes of magnetometer data and 52, 800 bytes of accelerometer data for each car that passed by them. Given this information, we had two key questions. The first is: what kind of backend infrastructure would be required to allow the system to perform acceptably if deployed on a city-wide or nation-wide scale? The second is: what is the best way of estimating the expected load and testing the backbone network? The answers to these questions are outside the scope of this article, while the questions prompted us to undertake the research that resulted in the development of Maestro.

### 6.2. Set-Up of the Simulated Scenario

The simulation was designed using the Symphony simulation framework. This allowed us to re-use code from the test site deployment described in Section 3. Each road segment contained 14 sensor nodes and one gateway and was simulated on a single simulation node. The gateway is a simulated device that was developed in ns-3. It communicates with other nodes and forwards sensor data to a real backbone system deployment. The backbone was also integrated into Amazon's AWS.

Sensory data generated by different passing cars was recorded in advance using the real-world system. This data was supplied to the Symphony's sensory model. During the simulations, the underlying node was reading sensory data according to the relative time stamp of the measurements.

These simulated sensory data were processed by the backend system and used to determine the type of car that had passed. The specific details of this scenario are beyond the scope of this article and will be presented in full elsewhere.

### 6.3. Going Big-The Affordable Cloud

Table 2 shows the cost of running a simulation of this type using different VM instances for a system with one million nodes. While the precise values presented in this table are only valid for this particular simulation, it is clear that performing simulations of this size using cloud systems is much more affordable than buying hardware with the necessary computational capacity. Note once again that it is more efficient to use more expensive, high performance instances to maintain the total cost on acceptable level.

The benefits of such large-scale simulations and the need to be able to perform them inexpensively have been discussed previously. However, a factor that has not previously been mentioned is that simulations of this sort generate immense quantities of data; the precise amount will depend on the details of the scenario and the nature of the data to be collected. Even though only four data cells were logged in this particular experiment (node_id, gateway, timestamp, data_size), a traffic intensity of one car passage every 5 min would create a 4 × 12, 000, 000 data set, corresponding to around 200 MB of data. It is thus clear that a much larger amount of data could very easily be generated during a simulation conducted with the aim of analyzing WSN performance in a holistic way.

## 7. Conclusions

The ongoing evolution of wireless sensor networks into complex systems that will play key roles in the development of cyber-physical systems and the Internet of Things has made the development, testing and validation of even small-scale WSN applications into challenging tasks that can easily consume significant resources. Simulations are vital for circumventing these problems, because they make it possible to test, verify and experiment with new technologies in a way that is repeatable and comparatively inexpensive. Therefore, as the size and complexity of WSNs and related systems increases, so too does the need for large-scale simulation tools that can be used to model their behavior.

The introduction of the Infrastructure as a Service paradigm has led to the availability of relatively cheap and scalable computational platforms that are accessible to a very wide audience that can be used to perform large-scale simulations in a credible and reproducible fashion and to easily share their results and setup conditions with anyone who may be interested.

This article introduce Maestro, an orchestration framework for large-scale WSN simulations. Maestro was designed to be a independent framework that enables the automated deployment of either large-scale simulations or many serial simulations in parallel. We have demonstrated its utility for benchmarking the performance of cloud systems and shown how it can be used to identify optimal VM instances for a given simulation. Moreover, we showcased its use in an ITS application and described the methodology used to set up the associated instances. It was further demonstrated that the size of the network that can be simulated using Maestro is limited only by the economic resources of the user. Our showcase simulation demonstrated that cloud computing services can be used to simulate a wireless sensor network with one million sensor nodes at a cost of only a couple of thousand dollars, which is substantially cheaper than performing similar experiments on dedicated hardware. While such simulations are much cheaper to perform than a real-world system would be to deploy, they can generate very large quantities of data. It is therefore important for the user to carefully consider their analysis strategies and to identify the key performance metrics that they will use before deploying the actual simulation.

The use of a public cloud service, such as Amazon Web Services, also allows researchers to easily share their full results and exhaustive information on the setup of their simulations with other members of the scientific community. It is also straightforward to create a virtual image that can be used to perform the simulation and to then make it publicly available simply by providing a link in the published article reporting the work. At current prices, it would cost less to store such an image than to buy one cup of coffee per month, so the storage cost is negligible. The results of the simulations and the data sets they generate can also be stored in this way and be made publicly available via cloud storage systems, such as S3 or Dropbox. This would obviate one of the current criticisms of simulation studies and create opportunities for cross-disciplinary research using open data sets and pre-configured simulation scenarios.

## Figures and Tables

**Figure 13.**
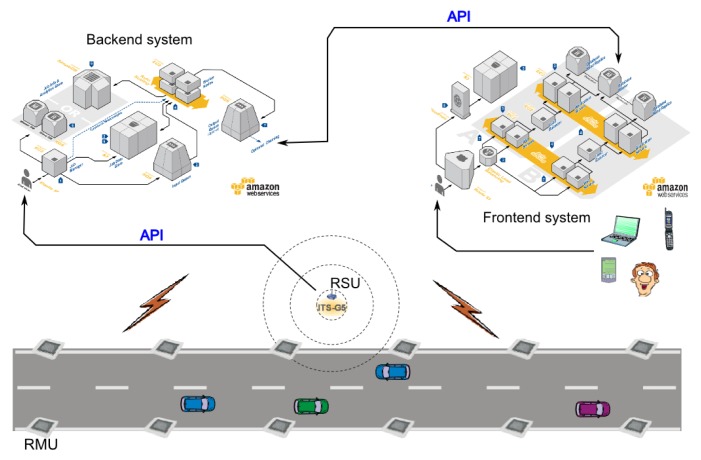
Incoming traffic load on the backend system as a function of the number of “islands”.

**Table 1.** Amazon AWS Instance comparisons. The Elastic Compute Unit (ECU) is an expression defining the amount of CPU that is allocated to a particular instance.

	**Type**	**Processor Arch**	**ECU**	**Price**
**Standard On-Demand Instances**				
Small	m1.small	32/64	1	$0.065
Medium	m1.medium	32/64	2	$0.130
Large	m1.large	64	4	$0.260
Extra Large	m1.xlarge	64	8	$0.520
**Second Generation Standard On-Demand Instances**				
Extra Large	m3.xlarge	64	13	$0.550
Double Extra Large	m3.2xlarge	64	26	$1.100
**Micro On-Demand Instances**				
Micro	t1.micro		≤2	$0.020
**High-Memory On-Demand Instances**				
Extra Large	m2.xlarge	64	6.5	$0.460
Double Extra Large	m2.2xlarge	64	13	$0.920
Quadruple Extra Large	m2.4xlarge	64	26	$1.840
**High-CPU On-Demand Instances**				
Medium	c1.medium	32/64	5	$0.165
Extra Large	c1.xlarge	64	20	$0.660

**Table 2.** Details on the cost and number of instances required to simulate an ITSnetwork with one million nodes based on the road surface network (RSN) system.

	**m1.small**	**m1.large**	**m3.xlarge**	**c1.xlarge**
**Intersection (1)**	1	3	16	16
**Total nodes per instance (2)**	14	28	224	224
**Cost per instance (3)**	$0.130	$0.260	$0.550	$0.660
**Normalized cost (5)**	2.08	1.387	0.550	0.660
**Number of instance required (6)**	71,429	23,810	4,099	4,099
**Total cost per hour (7)**	$9,286	$6,191	$2,255	$2,706
